# Procaspase-Activating Compound-1 Synergizes with TRAIL to Induce Apoptosis in Established Granulosa Cell Tumor Cell Line (KGN) and Explanted Patient Granulosa Cell Tumor Cells In Vitro

**DOI:** 10.3390/ijms22094699

**Published:** 2021-04-29

**Authors:** Powel Crosley, Anniina Farkkila, Adrianne L. Jenner, Chloé Burlot, Olivia Cardinal, Kyle G. Potts, Kate Agopsowicz, Marjut Pihlajoki, Markku Heikinheimo, Morgan Craig, Yangxin Fu, Mary M. Hitt

**Affiliations:** 1 Department of Oncology, University of Alberta, Edmonton, AB T6G 2E1, Canada; powel@ualberta.ca (P.C.); kyle.potts@ucalgary.ca (K.G.P.); kca@ualberta.ca (K.A.); yangxin@ualberta.ca (Y.F.); 2 Department of Obstetrics and Gynecology, University of Helsinki and Helsinki University Hospital, Haartmaninkatu 8, 00014 Helsinki, Finland; anniina.farkkila@helsinki.fi (A.F.); marjut.pihlajoki@helsinki.fi (M.P.); 3 Department of Mathematics and Statistics, Université de Montréal, Montréal, QC H3T 1J4, Canada; adrianne.jenner@qut.edu.au (A.L.J.); chloe.burlot@orange.fr (C.B.); olivia.cardinal@umontreal.ca (O.C.); morgan.craig@umontreal.ca (M.C.); 4 Sainte-Justine University Hospital Research Centre, Montréal, QC H3T 1C5, Canada; 5 Children’s Hospital, Pediatric Research Center, University of Helsinki and Helsinki University Hospital, Haartmaninkatu 8, 00014 Helsinki, Finland; markku.heikinheimo@helsinki.fi; 6 Department of Pediatrics, Washington University, St. Louis, MO 63130, USA

**Keywords:** caspase-3, GCT, ovarian cancer, PAC-1, mathematical modelling

## Abstract

Granulosa cell tumors (GCT) constitute only ~5% of ovarian neoplasms yet have significant consequences, as up to 80% of women with recurrent GCT will die of the disease. This study investigated the effectiveness of procaspase-activating compound 1 (PAC-1), an activator of procaspase-3, in treating adult GCT (AGCT) in combination with selected apoptosis-inducing agents. Sensitivity of the AGCT cell line KGN to these drugs, alone or in combination with PAC-1, was tested using a viability assay. Our results show a wide range in cytotoxic activity among the agents tested. Synergy with PAC-1 was most pronounced, both empirically and by mathematical modelling, when combined with tumor necrosis factor-related apoptosis-inducing ligand (TRAIL). This combination showed rapid kinetics of apoptosis induction as determined by caspase-3 activity, and strongly synergistic killing of both KGN as well as patient samples of primary and recurrent AGCT. We have demonstrated that the novel combination of two pro-apoptotic agents, TRAIL and PAC-1, significantly amplified the induction of apoptosis in AGCT cells, warranting further investigation of this combination as a potential therapy for AGCT.

## 1. Introduction

Granulosa cell tumor (GCT) is a malignant sex-cord stromal cell form of ovarian cancer that constitutes ~5% of ovarian neoplasms [1]. The majority (89%) of cases are early-stage GCT [2], yet they present a conundrum: although five-year survival is >90%, GCT is known for late recurrence and ~80% of women who relapse will die of the disease [1,3]. Surgery is the primary treatment modality and both European and American clinical guidelines recommend platinum-based therapies if chemotherapy is warranted [4,5]. However, multiple retrospective analyses of case histories have shown that there is no survival advantage between adjuvant chemotherapy, radiotherapy, and observation [6,7]. There are two forms of GCT, adult (AGCT) and juvenile GCT, with 95% of GCTs being AGCT [2]. In 2009, Shah et al., discovered a unique somatic mutation (FOXL2C134W) that was present in 97% of AGCT cases and in the only available AGCT-derived cell line, KGN [8].

Recently, there have been reports of drug screening studies involving AGCT (KGN and/or human tumor cells). A high-throughput study reported by Haltia et al. demonstrated AGCT sensitivity to the tyrosine kinase inhibitor dasatinib and mTOR inhibitor everolimus that act synergistically with the microtubule-targeted chemotherapeutic agent paclitaxel [9]. Roze et al. examined responses of AGCT patient-derived cell cultures to 11 monotherapies and 12 combination therapies. None of the monotherapies tested were very effective, but their study indicates a potential for combination of carboplatin, paclitaxel, and the kinase inhibitor alpelisib for treating this disease [7].

We have taken an alternate approach to examining novel combinations of drugs for treating AGCT, by focusing on facilitating apoptosis of the tumor cell. One of the main mechanisms of tumor cell killing by anti-cancer drugs is induction of programmed cell death, apoptosis. Apoptosis is activated in response to irreparable DNA damage, external stimuli, or other cellular stresses, with apoptotic pathways ultimately converging on the activation of procaspase-3 to caspase-3, the primary effector of apoptosis. Many cancers highly express procaspase-3 and balance that with inhibitors of caspase-3 in order to survive [10]. Caspase-3 activity is also regulated by Zn^2+^, which reportedly has three binding sites in caspase-3 [11]. While the full impact of multiple binding sites is unclear, it is thought that Zn^2+^ inhibits access to the active site (near His121) and may interfere with access to the inter-subunit cleavage site Ile-Glu-Thr-Asp175 (IETD^175^) (Figure S1) [11,12]. Procaspase activating compound-1 (PAC-1) is a small-molecule compound that was identified through a high-throughput screen of ~20,500 small-molecule compounds for the ability to activate procaspase-3 in vitro [10]. Later, it was determined that PAC-1 prepares procaspase-3 for activation by sequestering labile inhibitory Zn^2+^ ions from the zymogen, allowing it to undergo either auto-maturation into an active state or cleavage by initiator caspases, caspase-8 and caspase-9 [13]. The ability of caspase-3 to auto-mature means that sequestration of Zn^2+^ potentiates activation of apoptosis even when upstream signals are defective, in a manner directly proportional to the concentration of procaspase-3 in the cell, explaining PAC-1 selectivity for cancer [10]. PAC-1 has shown efficacy as an anti-cancer agent in vitro and in vivo, and has minimal activity towards other zinc-dependent enzymes [14], which may account in part for its demonstrated safety. PAC-1 is under investigation in phase I trials for advanced malignancies (NCT02355535, NCT03332355).

Tumor necrosis factor-related apoptosis inducing ligand (TRAIL) is a transmembrane protein that binds death receptor-4 or -5 (DR4/DR5) triggering the extrinsic apoptotic pathway as well as the intrinsic pathway via caspase-8 truncation of BID [15,16]. TRAIL induces apoptosis in cancer cells without toxicity to normal cells and clinically has been well-tolerated by patients [17,18,19]. Perplexingly however, clinical trials with soluble TRAIL have failed to display efficacy [20]. Potential limiting factors include a short half-life in vivo, inability to reach therapeutic concentration at the tumor site, and downregulation of the death receptors or of downstream mediators of apoptosis [21].

Interestingly, normal granulosa cells and most GCTs express DR4/5 [1,22] and the GCT model cell line KGN is reported to be sensitized to TRAIL in vitro when combined with cisplatin, proteasome inhibitors, or mitochondrial membrane potential uncouplers [23,24]. Here, we report that procaspase-3-activator PAC-1 synergizes with TRAIL to kill both KGN cells and four independent patient-derived GCT cultures, but not normal cells, via apoptosis induction. We also report that PAC-1/TRAIL combination appears to be more effective than PAC-1 combined with chemotherapy drugs carboplatin or gemcitabine, or with embelin, an inhibitor of the X-linked inhibitor of apoptosis protein (XIAP), without increasing sensitivity of normal cells. We propose that the PAC-1/TRAIL combination should be further investigated as a treatment option for patients with GCT.

## 2. Results

### 2.1. Granulosa Cell Tumor Cells Display Sensitivity to PAC-1 and Other Select Apoptosis-Inducing Agents

**Figure 1:**
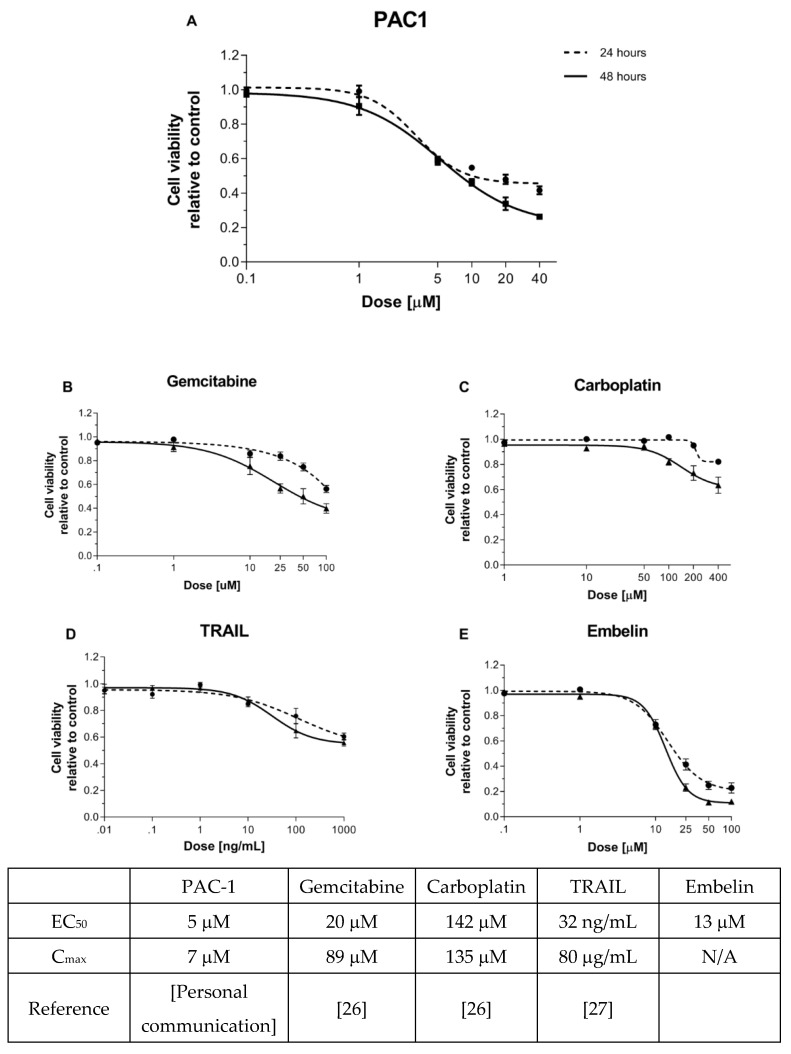
KGN cells are more sensitive to caspase-activating compounds than to upstream apoptotic agents. KGN cells (7000/well) were seeded into 96-well plates containing (**A**) PAC-1 (0–40 µM), (**B**) gemcitabine (0–100 µM), (**C**) carboplatin (0–400 µM), (**D**) TRAIL (0–1000 ng/mL), or (**E**) embelin (0–100 µM). Viability was determined 24 and 48 h later by resazurin metabolic assay. Points represent mean +/− standard error of the mean (SEM) for 3–4 independent experiments. EC_50_ values in the table reflect values calculated from this experiment. C_max_ values reflect the maximum concentrations of the indicated drugs in the serum of treated patients as reported in the literature (references as noted).

Similar to many other tumor cells [7], the only available AGCT cell line, KGN, expresses appreciable levels of caspase-3 (Figure S2). We examined whether this property could be exploited to induce KGN cell killing by treating with increasing concentrations of PAC-1 up to 40 µM (Figure 1A). At doses higher than this, PAC-1 is reported to induce killing independent of procaspase-3 and caspase-3 [25]. This dose–response assay for viability, confirmed by visual inspection of the cell cultures, demonstrated PAC-1 sensitivity of KGN cells. Cytotoxicity appeared to be at least partially dependent on caspase-3, as killing was reduced in KGN cells transfected with an shRNA targeting the caspase-3 gene compared to KGN cells transfected with a control shRNA (Figure S2).

We next compared the response of KGN cells to PAC-1 with responses to a panel of other apoptosis-inducing agents that act upstream of caspase-3, the target of PAC-1. Conventional chemotherapeutic agents, namely carboplatin (alkylating agent) and gemcitabine (nucleoside analog), interfere with DNA replication, activating the DNA damage response [24] and subsequent intrinsic apoptosis. In contrast, TRAIL binds cell death receptors and induces caspase-3 activation through both intrinsic and extrinsic apoptotic pathways. Embelin is a SMAC-mimetic that inhibits XIAP, an inhibitor of caspase-3. Clinically relevant drug doses were selected for this study, based on either established dosing guidelines or results from ongoing clinical trials, with viability assessment at 24 and 48 h [26,27].

The most potent of the drugs tested, based on the dose at which 50% cell death was induced, were PAC-1 and embelin, followed by gemcitabine, with carboplatin and TRAIL failing to reach 50% killing in this assay (Figure 1A–E). The results with gemcitabine and carboplatin are consistent with clinical observations showing poor efficacy of standard chemotherapy in treating AGCT [28,29]. The lack of response to TRAIL was unexpected, as KGN expresses the TRAIL receptor DR5 (Figure S3). Interestingly, the drugs that induced the greatest cytotoxicity act at the most downstream point of apoptosis, where the intrinsic and extrinsic pathways converge. Furthermore, the most potent drugs act proximally to procaspase-3, which is highly overexpressed in KGN cells (Figure S2). These results suggest that procaspase-3 activation could be an effective therapeutic strategy for AGCT.

### 2.2. PAC-1 Displays No or Low Synergy with Carboplatin, Gemcitabine or Embelin, But Strong Synergy with TRAIL in Killing KGN Cells

PAC-1 has been shown to display synergistic killing with standard chemotherapy drugs in several cancers [13]. A synergistic relationship between drugs allows the use of lower drug doses with potentially less off-target toxicity [30]. PAC-1 synergy in GCT was tested by assessing viability of KGN cells treated with PAC-1 in combination with gemcitabine, carboplatin or embelin. The rationale for testing these combinations was to determine whether they could synergize in inducing apoptosis by targeting the pathway at different levels. Drug interaction was calculated using both the Loewe and Bliss reference models. The Bliss model uses a probabilistic approach assuming that the two drugs respond independently: suitable for compounds that target different pathways. The Loewe model compares the dose response of the individual compounds to the response of the combination, assessing deviations from additivity: most applicable when drugs have a similar mode of action on the same pathway or target [31].

**Figure 2:**
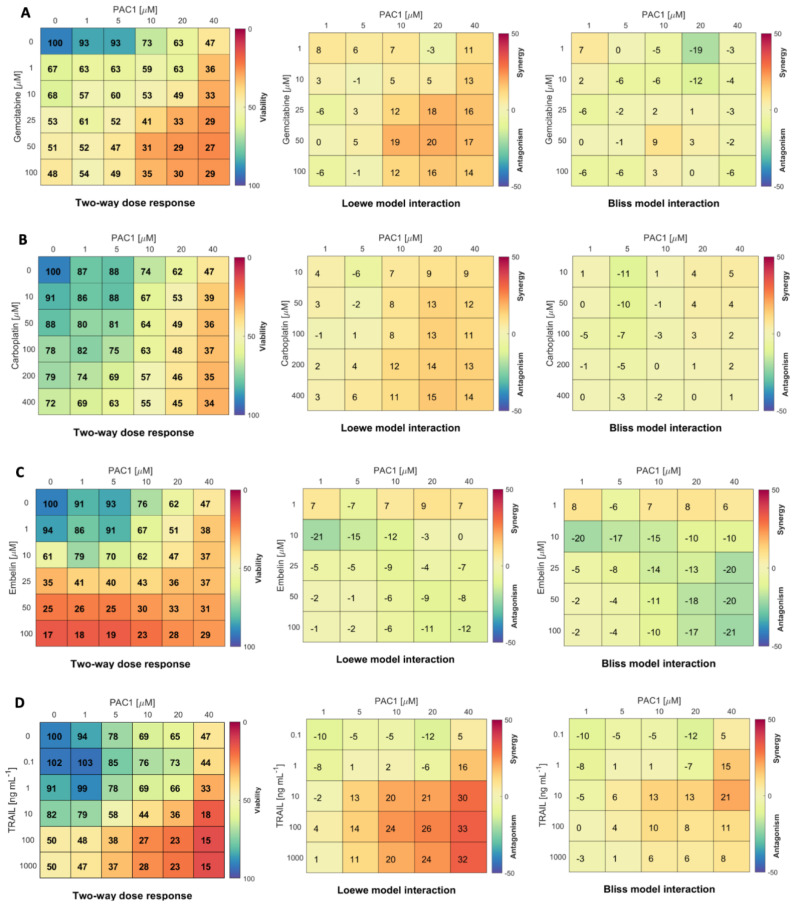
PAC-1 is more synergistic with TRAIL than with gemcitabine, carboplatin or embelin in KGN cells. Two-way dose–response assays were set up with 5000 KGN cells/well in 96-well plates. Wells were treated for 48 h with PAC-1 doses from 0 to 40 µM combined with (**A**) gemcitabine (0–100 µM); (**B**) carboplatin (0–400 µM); (**C**) embelin (0–100 µM); or (**D**) TRAIL 0–1000 ng/mL. Viability was determined by resazurin metabolic assay (**left panels**, % viability relative to untreated control wells is indicated by color gradient). Drug interaction was calculated by Matlab module Combenefit using both the Loewe (**centre panels**) and Bliss (**right panels**) reference models [32] (*n* = 3 independent experiments).

We observed that combinations of PAC-1 with gemcitabine, carboplatin or embelin were not highly effective in boosting KGN killing (Figure 2 [32]). Gemcitabine combined with PAC-1 induced moderate loss in viability with low to moderate synergy (Loewe analysis) or very little synergy (Bliss analysis). Carboplatin, which induced low to moderate cytotoxicity in combination with PAC-1, showed low or no synergy. Similar results were seen with PAC-1 in combination with radiation (Figure S4), another inducer of the DNA damage response [33]. Embelin, although cytotoxic on its own and in combination with PAC-1, displayed a weakly antagonistic interaction.

As a single agent, TRAIL treatment did not induce high levels of KGN killing (Figure 1D). However, reports that other agents can sensitize cancers to TRAIL [24,34], and the fact that TRAIL is well-tolerated in clinical trials [17,19], stimulated us to investigate whether TRAIL could be more effective in combination with PAC-1. Two-way dose–response assays combining TRAIL and PAC-1 to treat KGN cells (Figure 2D) showed an increase in loss of viability and strong synergy with the combination. Z-scores from two-way dose–response data were used to determine PAC-1 and TRAIL dose combinations that were significantly different from untreated (Figure S5A). Z-scores, together with PAC-1 EC50 values (Figure S5B) prompted us to select a PAC-1 dose of 20 µM to use in combination with the relatively low dose of 10 ng/mL TRAIL as a standard treatment in subsequent studies.

Since most toxicity in patients is the result of off-target effects of drugs on normal tissues, we investigated the impact of PAC-1 and TRAIL on a human dermal fibroblast cell line (F202) as a surrogate for non-replicating, non-cancerous tissue. In the two-way dose–response viability assay, F202 cells were much less sensitive to PAC-1/TRAIL combinations than KGN cells were, even at relatively high doses of PAC-1 (Figure S6A). Similar results were seen with a second fibroblast cell line and a cell line cultured from normal human kidney cells (Figure S6B).

### 2.3. Using Mathematical Modelling to Validate Synergy of PAC-1 and TRAIL and Optimal Dosages

**Figure 3:**
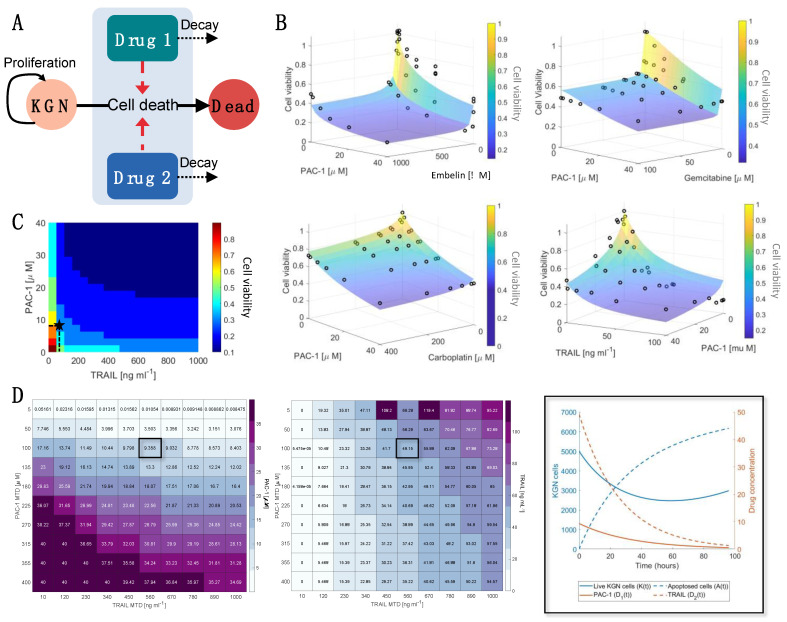
Mathematical modelling confirms synergy of PAC-1 and TRAIL and determines optimal drug combination. (**A**) We developed a mathematical model for the effect of drug combinations on live proliferating KGN cells (K(t)). The pharmacokinetics of each drug were modelled using a linear decay term, and the combined drug-induced cell death rate $E\left(D_{1},\;D_{2},\;\;\Psi \right)$ was modelled as dependent on the synergistic (0≤Ψ<1), additive (Ψ=1), or infra-additive (Ψ>1) interactions between the drugs (Equations (2)–(8) in Materials and Methods). (**B**) Values for Ψ were obtained by fitting the model in (A) to the cell viability measurements (black circles) and are noted in Table 1 along with the corresponding model approximations as transparent surface plots. (**C**) The model’s predicted cell viability for varying PAC-1 and TRAIL dosages is denoted through shaded regions corresponding to increments of 0.1 in the cell viability. (**D**) The dosage of PAC-1 (**left**) and TRAIL (**middle**) that minimized cell viability and overall drug concentration was determined using a genetic algorithm. This was determined for varying PAC-1 and TRAIL maximum tolerated dose (MTD) concentrations. For a maximum tolerated PAC-1 dose of 50 μM and TRAIL dose of 560 ng/mL, the resulting model prediction for KGN live and dead cells and PAC-1/TRAIL concentrations is plotted (**right**) and noted on (**C**) as a black star. Enlarged versions of Figure 3D tables are provided in Figures S9 and S10. The corresponding optimal cell viability for each PAC-1 and TRAIL MTD is in Figure S11.

Mathematical models are increasingly leveraged to quantify drug efficacy and predict optimal therapeutic strategies [35,36]. To that end, we developed a mathematical model for the induction of apoptosis in KGN cells by gemcitabine, carboplatin, TRAIL, embelin and PAC-1 (Figure 3A). The proliferation rate of KGN cells was determined through fitting an exponential growth rate (Equation (1) in Materials and Methods) to the KGN cell count measurements by Imai et al. [37] (Figure S7). Using data from the single dose–response curves (Figure 1) the half-effect, $IC_{50}$, for each drug and drug-induced death rate, were determined through hierarchical fitting (Equations (1)–(8) in Materials and Methods; results in Figure S8 and Table S1). Lastly, the drug interaction potency Ψ was then obtained through fitting the two-way drug-response measurements (from Figure 2) to the model (Figure 3B and Table 1).

Resulting drug-interaction strengths, *Ψ*, obtained for each drug combination validated the predictions from the in vitro dose–response experiments as we found a strong synergistic relationship exists between PAC-1 and TRAIL (*Ψ* = 0.8). For all other drug combinations, we estimated that an infra-additive (or antagonistic) relationship existed between those drugs (Table 1). We also found significant reductions in cell viability for PAC-1 and TRAIL, and PAC-1 and embelin. The combined strength of PAC-1 and embelin is more likely due to the individual potency of embelin and PAC-1 (Figure 1 and Figure S8) given their antagonistic interactions (*Ψ* = 1.4).

To further investigate the two-way dose–response effect of PAC-1 and TRAIL, we determined the cell viability in 0.1 increments for varying PAC-1 and TRAIL concentrations (Figure 3C). Despite the synergistic relationship between PAC-1 and TRAIL, for very low concentrations there is little-to-no effect on the cell viability (predicted viability > 0.75). However, increasing TRAIL concentrations slightly (>10 ng/mL) resulted in a dramatic change in cell viability at 48 h. In contrast, a more gradual impact on cell viability is seen with increasing PAC-1 concentration. This is further illustrated through comparing the relative change in cell viability to dosage increments (Figure S11) which showed that there was minimal benefit to increases in PAC-1 or TRAIL given TRAIL concentrations over 50 ng/mL. Overall, once the TRAIL concentration exceeds 50 ng/mL cell viability is not greatly affected by the concentration of either drug, potentially due to saturation of the TRAIL receptor.

Using the calibrated model, we then determined the optimal dosage size for PAC-1 and TRAIL using a genetic algorithm (Figure 3D). Genetic algorithms are heuristic global optimization routines inspired by natural selection that can be used to optimize dosage protocols [36,38]. In this optimization, we minimized both the cell viability and drug concentration relative to the maximum tolerated dose (MTD) for each drug. For varying MTD, the optimal combinations of PAC-1 and TRAIL were determined along with the resulting cell viability (Figure S11). From this analysis, we determined ranges of PAC-1 and TRAIL that are optimal for a range of MTD. PAC-1 dosages from 10 μM–40 μM combined with TRAIL dosages from 5 to 60 ng/mL were able to result in minimal cell viability (~0.2–0.3) and minimal drug toxicity.

### 2.4. Combining TRAIL with PAC-1 Rapidly Induces Caspase-3 Activity in KGN Cells

**Figure 4:**
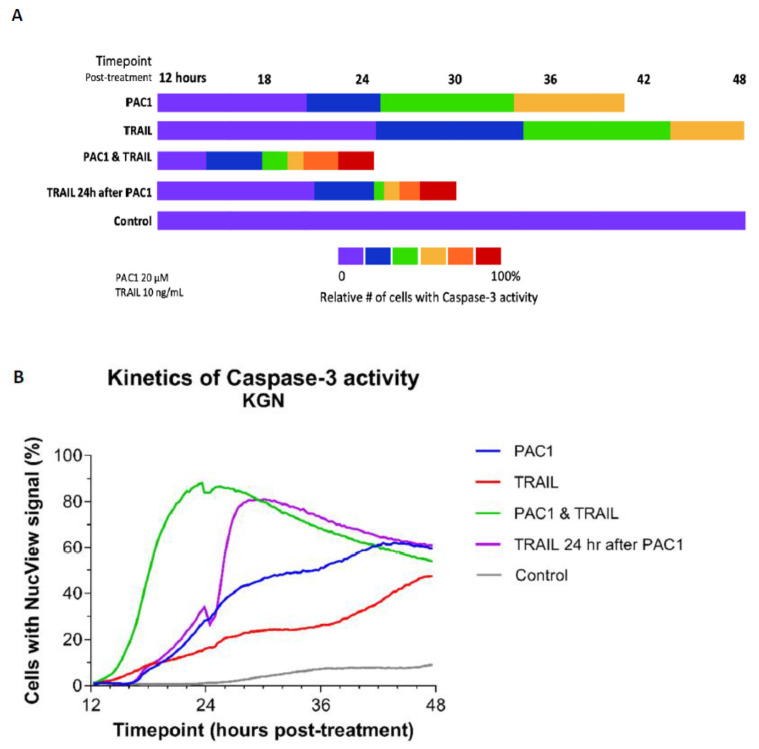
Combining PAC-1 with TRAIL induces rapid activation of caspase-3 in KGN cells. KGN cells (5000/well) were treated with either 20 µM PAC-1, 10 ng/mL TRAIL, or 20 µM PAC-1 combined with 10 ng/mL TRAIL. Cells were then stained with Hoechst 33,342 and NucView-488 Caspase-3 substrate and monitored over 48 h for caspase-3 cleavage (Molecular Devices ImageXpress high-content imaging). (**A**) Imagery was then processed using the MetaXpress Multi-wavelength Cell Scoring module and the number of NucView-fluorescent cells was determined relative to the total number of Hoechst-stained cells (percentages are color-coded as indicated in the scale). Total length of bars indicates time to reach maximal NucView-fluorescence. Data shown are representative of multiple analyses of caspase-3 activity. (**B**) The percentage of cells/well expressing caspase activity is plotted for each drug condition. Note that maximal activity was measured in the PAC-1/TRAIL combination wells at 24 h while adding TRAIL 24 h after treatment with PAC-1 maximal activity was reached within 3 h. Destruction of apoptotic cells at this time point may explain reduced percentage of NucView + cells after 24 h. In contrast, maximal activity in the single agent wells was at 44 h or later.

The kinetics of PAC-1-TRAIL induction of caspase-3 activity in KGN cells was tracked over time using high-content imaging (Molecular Devices ImageXpress, Molecular Devices, LLC., San Jose, CA, USA) (Figure 4). PAC-1 (20 µM) generated a moderate level of caspase activity by 36 h, similar to results of the PAC-1-induced loss of viability assay (Figure 1A). TRAIL alone (10 ng/mL) was less active than PAC-1 in both cytotoxicity (Figure 1D) and procaspase-3 activation. However, the combination of PAC-1 and TRAIL rapidly and dramatically amplified the proportion of active-caspase-positive cells (Figure 4) reaching over 80% by 24 h (Figure 4B). Western blot analysis (Figure S12) demonstrated an increase in caspase-9 cleavage with combined PAC-1 and TRAIL relative to single agents. Cleaved caspase-3 levels were also higher with the combination than in cells treated with PAC-1 alone, although not significantly higher than with TRAIL alone. It should be pointed out that the increased rate of apoptosis with combination treatment makes it more challenging to capture cells after caspase-3 cleavage but before cells have undergone complete apoptosis and are no longer intact. Due to this recovery issue, the high content imaging results are likely to be more reliable than the results of Western blotting.

Perhaps even more striking was administration of TRAIL starting 24 h after PAC-1 treatment was initiated. Under these conditions, maximal activity was achieved within 4–5 h of TRAIL administration (Figure 4B). This suggests that PAC-1 may prime procaspase-3 molecules for activation, which then enhances TRAIL-induced death signaling for execution of caspase-mediated apoptosis.

### 2.5. Combining TRAIL with PAC-1 Reduces Proliferation and Increases Caspase-3 Activity in Patient-Derived Granulosa Cell Tumor Cells

**Figure 5:**
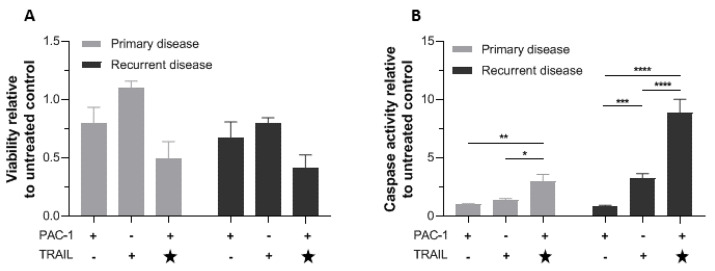
PAC-1 combined with TRAIL reduces viability and increases caspase-3 activity in cultured patient-derived GCT cells. GCT cells explanted from 4 patients (primary tumor samples from 2 patients and recurrent tumor samples from the other two patients) were cultured separately for 5 days then treated with 20 µM PAC-1 (48 h), 10 ng/mL TRAIL (48 h), or 20 µM PAC-1 (24 h) followed by a second 24-h treatment with 20 µM PAC-1 plus 10 ng/mL TRAIL (. (**A**) Viability at endpoint measured by WST-1 assay. (**B**) Caspase-3 activity at endpoint measured with Caspase-Glo^®^ 3/7. Star (★) indicates that TRAIL was added 24 h after PAC-1 treatment. Responses relative to untreated cells are shown. Bars represent mean response +/− SEM, for samples from the two patients with primary disease separately from the two patients with recurrent disease. Significance tested by two-way ANOVA (* *p* < 0.05; ** *p* < 0.01; *** *p* < 0.001; **** *p* < 0.0001).

The potential clinical utility of PAC-1/TRAIL combination treatment was investigated in vitro using patient-derived explants from primary and recurrent AGCT. To minimize alterations in the GCT phenotype, explants were used fresh, not frozen, and cultured only 5 days in vitro before the analysis was conducted. Our in vitro work with KGN cells suggested that addition of TRAIL 24 h after initiation of PAC-1 treatment induced rapid and efficient activation of caspase-3 (Figure 4), so for this experiment TRAIL was added to PAC-1 treated cells halfway through the 48 h cell viability assay (Figure 5). In cultures of both primary and recurrent disease, the combination of PAC-1 followed by TRAIL resulted in measurable loss of cell viability relative to single agents and to untreated controls, and significant increase in caspase-3 activity with primary and recurrent disease treated with TRAIL alone or TRAIL after PAC-1 (*p* < 0.05). Interestingly, it appeared that recurrent disease had greater activation of caspase-3 than primary disease, with at least similar, if not more, induced cell killing.

## 3. Discussion

Granulosa cell tumor (GCT) is a rare, sex-cord stromal neoplasm and research on new therapies is challenging due to the paucity of research tools: only one established cell line (KGN); the lack of an animal tumor model carrying the characteristic FOXL2C134W mutation; and few clinical trials owing partly to the difficulty in recruiting enough participants (e.g., NCT02101684). Clinically, common chemotherapy agents are generally ineffective for GCT [28,29,39] and the lack of effective treatment options contributes to the ~80% mortality among women who suffer relapse [40]. Though GCT has been considered a hormonally responsive tumor, trials using agents interacting with the ovarian steroidogenesis pathway have had inconclusive results [41].

We sought to determine whether a different approach using a novel combination of compounds would induce apoptosis without the toxicity observed with many standard chemotherapies. We found that the procaspase-3 activator PAC-1 was cytotoxic to KGN cells, and this activity was at least partly caspase-3-dependent. KGN cells were also sensitive to embelin, a SMAC-mimetic that inhibits the endogenous caspase-3 inhibitor XIAP; but were less sensitive to other apoptosis-inducing agents including carboplatin, gemcitabine, and TRAIL. It would be interesting in future studies to investigate whether SMAC mimetics that target other IAPs may also have activity against GCT. KGN appears therefore to be more sensitive to agents that influence caspase-3 directly than to agents that act upstream in apoptotic signaling pathways.

To examine potential benefits from drug combination, we paired PAC-1 with each of the other single agents. Interestingly, the combination of TRAIL and PAC-1 was the only combination that displayed strong synergy and was very effective at reducing viability of KGN cells. It would be informative to repeat this drug screen using patient samples, however availability of patient tissue is problematic. Mathematical modelling was consistent with our experimental synergy analyses, and validated the concentrations of TRAIL and PAC-1 used in subsequent experiments. Non-cancerous cells were much less sensitive to PAC-1/TRAIL combination in agreement with published reports on the safety of PAC-1 and TRAIL individually [14,17,18]. Using the calibrated model, we predicted dosage regimes that optimize TRAIL and PAC-1 combination therapy for unique values of the MTD of either drug, helping to inform reliable clinical protocols.

Two other notable drug screens designed to identify new agents for treating AGCT have recently been reported. One study examined cytotoxicity of 11 different chemotherapeutic, anti-hormonal, and targeted drugs alone and in combination using a panel of 12 patient-derived AGCT cultures and KGN cells [9]. Similar to our study with KGN cells, Roze et al. found carboplatin to have low efficacy as a single agent, however, carboplatin was the only drug common to both our study and theirs. A comprehensive investigation by Haltia et al. examined selective cytotoxicity of 230 anti-cancer drugs using seven patient-derived AGCT cultures, KGN cells, and normal human granulosa and bone marrow cells as control in a high throughput assay [8]. Interestingly, this screen tested several apoptotic modulators, including inhibitors of Bcl2, Mdm2 and survivin (an IAP), as well as carboplatin and gemcitabine. The survivin inhibitor and one of the Bcl-2 inhibitors were among the top 15 AGCT-selective drugs, although the authors focused the remainder of their study on the tyrosine kinase inhibitor dasatinib and mTOR inhibitors. Not surprisingly, carboplatin and gemcitabine had variable/low activity and selectivity, consistent with our results. This study strengthens the hypothesis that apoptosis could be manipulated as a potential treatment for AGCT.

**Figure 6:**
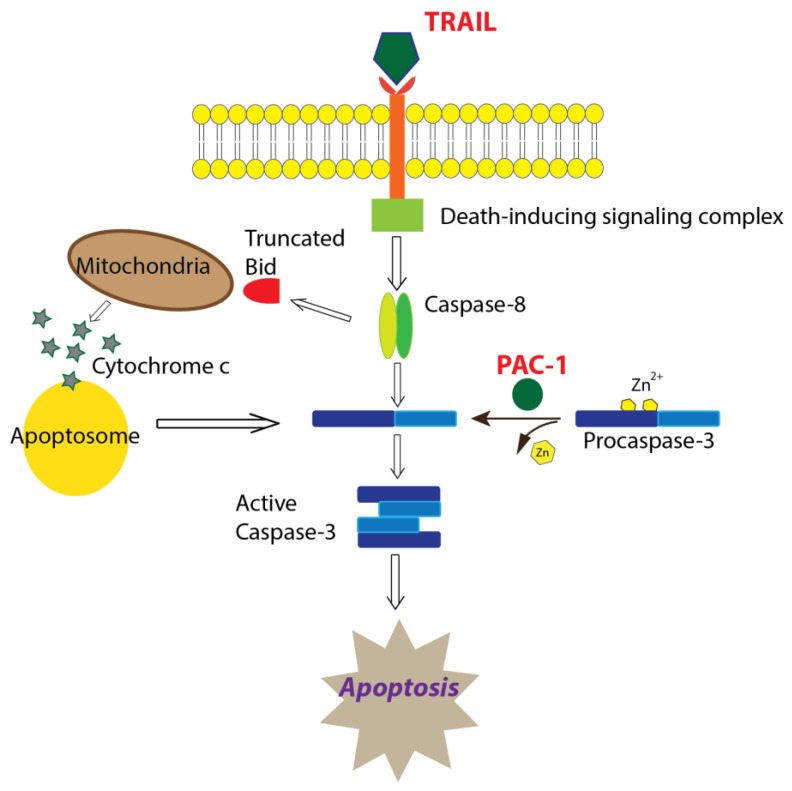
Proposed mechanism of apoptosis induction mediated by PAC-1 in combination with TRAIL. Based on data presented in this paper, we hypothesize that PAC-1 and TRAIL synergistically function to increase apoptosis through PAC-1 removal of inhibitory Zn^2+^ ions resulting in primed caspase-3 molecules, which are then activated through TRAIL-induced death-signaling that utilizes the extrinsic and/or intrinsic apoptotic pathway.

Using clinically relevant doses of PAC-1 and TRAIL, we established the kinetics of procaspase-3 activation in KGN cells. We observed a dramatically rapid increase in caspase-3 activity with the combination compared to agents alone, consistent with PAC-1—TRAIL synergistic cell killing. At these clinically relevant doses, PAC-1 did not increase the expression of DR5 (Figure S3D), and neither PAC-1 nor TRAIL alone induced a strong loss of cell viability. Therefore, we hypothesize that PAC-1, as a single agent, prepares procaspase-3 for activation in KGN cells but is not induced to carry out immediate auto-cleavage, and TRAIL, as a single agent, activates death signaling but this is muted by Zn^2+^ inhibition of procaspase-3/caspase-3. Furthermore, we propose that in combination, PAC-1 primes procaspase-3 to a ready-state and TRAIL sends the signal to spark the observed caspase-3 activity resulting in cell death (Figure 6).

Finally, we examined the effect of PAC-1 and TRAIL on patient-derived explants of GCT tumors as an indicator of clinical relevance. Due to the rarity of GCT (the incidence being approximately five cases/year in Helsinki), our study was limited to four patient cell cultures. Nonetheless, the results were consistent with the KGN-based assays in terms of the superiority of PAC-1/TRAIL combination and suggest that the combination might be active against both primary and recurrent disease, however this should be tested in a larger sample size.

TRAIL is a well-documented anti-cancer agent and is often efficacious in drug combination in vitro, although it has been less promising in clinical trials [15] due to a short half-life, ineffective dosing at the tumor site, and TRAIL resistance. Even so, during a phase I clinical trial of an agonistic monoclonal antibody targeting DR5 (PRO95780), a 57-year-old woman with AGCT received eight doses (4 mg/kg) of drug and showed a 23% reduction in measurable disease. The patient was followed for an additional 21 months without systemic therapy and with no evidence of progression [42]. We found that it is possible to increase the ability of TRAIL to induce apoptosis in GCT at very low doses by combining it with PAC-1, which we suggest primes procaspase-3 for activation by TRAIL-induced death signaling (Figure 6). Furthermore, we are currently developing a recombinant vaccinia virus gene therapy vector to deliver TRAIL directly to the tumor, which may overcome some of the limitations reported for clinical use of TRAIL, such as short half-life and ineffective dosing at the tumor site.

This novel combination of PAC-1 and TRAIL, which have both displayed low toxicity in clinical trials [17,18,19], appears effective in vitro warranting further preclinical development.

## 4. Materials and Methods

### 4.1. Cell Culture and Reagents

The human GCT cell line KGN (Riken BioResource Research Center, Ibaraki, Japan) was cultured in Dulbecco’s modified Eagle’s medium/nutrient mixture F12 (DMEM/F12, Sigma-Aldrich, St. Louis, MO, USA) with 5% fetal bovine serum (FBS) (Gibco, Waltham, MA, USA). Normal human fibroblast cells (F202 and N60, generously provided by Ted Tredget, University of Alberta) and normal human kidney cells (NKC, isolated from the proximal tubule, provided by Ron Moore) were cultured in DMEM/high glucose (Sigma-Aldrich) with 10% FBS. All culture media were supplemented with 2 mM L-glutamine, 100 U/mL penicillin, and 100 U/mL streptomycin) (Gibco). Cell line authentication was performed for KGN using short tandem repeat DNA profiling (Promega GenePrint 10 System, Madison, WI, USA) at the Genetic Analysis Facility at the Centre for Applied Genomics of The Hospital for Sick Children (Toronto, ON, Canada).

PAC-1 was generously provided by Paul Hergenrother (University of Illinois) and Hoechst 33,342 was generously provided by Linda Pilarski (University of Alberta). Other chemicals include recombinant human TRAIL (sTRAIL/Apo2L, Peprotech #310-04); carboplatin (Enzo Life Sciences #400-041); NucView 488 Caspase-3 substrate (Biotium #30029); and resazurin sodium salt (R7017), embelin (E1406), and gemcitabine hydrochloride (G6423-10) from Sigma-Aldrich.

### 4.2. Cell Viability/Metabolism Assay

For all cultures except patient-derived GCT cultures (for those see Section 4.4), 5000 cells/well were seeded in triplicate wells of a 96-well plate containing a range of doses of selected compounds and incubated at 37 °C with 5% CO_2_ for the time indicated. Resazurin (final concentration 44 µM) was then added to the medium and cells incubated up to four h at 37 °C. The reduction of resazurin was measured on a BMG FLUOStar Omega microplate reader (544 nm excitation/590 nm emission, BMG Labtech, Ortenberg, Germany). Blank-corrected relative fluorescence units (RFUs) were normalized to untreated control wells. Each assay was repeated at least 3 times.

### 4.3. Caspase-3 Activity Assay (KGN Cells)

Five thousand KGN cells/well in black 96-well plates were stained with 0.1 µg/mL Hoechst 33,342 and 1 µM NucView-488 Caspase-3 substrate for 30 min in the dark at 37 °C. Cells were then treated with 20 µM PAC-1, 10 ng/mL TRAIL, or PAC-1 combined with TRAIL and incubated at 37 °C for 12 h. Plates were then transferred to a Molecular Devices ImageXpress high-content screening instrument and incubated at 37 °C with 5% CO_2_. Images were acquired at four sites per well every 30 min until 48 h post-treatment. Imagery was processed using the MetaXpress Multi-wavelength Cell Scoring module and the number of NucView-fluorescent cells (using the FITC channel) were normalized to the number of Hoechst-stained cells (using the DAPI channel).

### 4.4. Patient-Derived GCT Cultures and Assays

Four patient-derived tumor samples, two from patients with primary disease and two from patients with recurrent disease, were collected by A. Farkkila and M. Pihlajoki with informed consent and in accordance with The Code of Ethics of the World Medical Association (Declaration of Helsinki, Ferney-Voltaire, France). All four samples were verified to possess the 402C → G (C134W) mutation in FOXL2. Tumors were processed as previously described [43]. Briefly, each fresh GCT tissue sample was minced then dissociated with 0.5% collagenase (Sigma–Aldrich) in DMEM/F12 (without FBS) for 2 h. The resulting cell suspension was filtered through a 140 µm mesh to obtain single cells, washed with PBS, resuspended in DMEM/F12 containing 10% FBS, then plated. After 5 days in culture, cells were treated with drugs as indicated. Viability was assessed using the cell proliferation agent WST-1 (Roche, Basel, Switzerland), and caspase activity was measured using the Caspase Glo^®^ 3/7 kit (Promega, Madison, WI, USA).

### 4.5. Mathematical Model for Drug Induced KGN Cell Apoptosis

KGN cells (K(t)) were assumed to be proliferating at a rate r, with the concentration of two drugs ($D_{1}\left(t\right)$ and $D_{2}\left(t\right)$) decaying at drug-specific rates $\kappa_{1}$ and $\kappa_{2}$. Each drug was modelled as inducing apoptosis in KGN cells to create dead (or apoptotic) cells (A(t)). The changes in KGN cells and drug concentrations are given by

(1) $$\frac{dK}{dt}=rK\left(t\right)-E\left(D_{1}\left(t\right),D_{2}\left(t\right),\Psi \right)K,$$

(2) $$\frac{dA}{dt}=E\left(D_{1}\left(t\right),D_{2}\left(t\right),\Psi \right)K,$$

(3) $$\frac{dD_{1}}{dt}=-\kappa_{1}D_{1},$$

(4) $$\frac{dD_{2}}{dt}=-\kappa_{2}D_{2},$$

where

(5) $$E\left(D_{1}\left(t\right),D_{2}\left(t\right),\Psi \right)=\frac{\delta_{1}I_{max,1}\beta_{1}\left(t\right)+\delta_{2}I_{max,2}\beta_{2}\left(t\right)+\left(\delta_{1}I_{max,1}+\delta_{2}I_{max,2}-\;\overset{¯}{\delta }I_{max,1}I_{max,2}\right)\beta_{1}\left(t\right)\beta_{2}\left(t\right)}{\beta_{1}\left(t\right)+\beta_{2}\left(t\right)+\beta_{1}\left(t\right)\beta_{2}\left(t\right)+1},$$

(6) $$\beta_{1}\left(t\right)=\frac{D_{1}{\left(t\right)}^{\gamma_{1}}}{{\left(\Psi IC_{50,1}\right)}^{\gamma_{1}}},$$

(7) $$\beta_{2}\left(t\right)=\;\frac{{\left(\xi D_{2}\left(t\right)\right)}^{\gamma_{2}}}{{\left(\Psi IC_{50,1}\right)}^{\gamma_{2}}},$$

(8) $$\xi =\frac{IC_{50,1}}{IC_{50,2}},$$

and the relevant drug $I_{max}$ and $IC_{50}$ are denoted by a subscript (i.e., $I_{max,1},\;I_{max,2}$ and $IC_{50,1},\;IC_{50,2}$). The rate of drug induced KGN cell apoptosis is given by $\delta_{1}$ and $\delta_{2}$ for each drug, respectively, with $\overset{¯}{\delta }$ representing interactions in the dual drug case, and the ratio of potency of each drug by ξ. The potency term Ψ is a measure of drug interactions. Ψ=1 indicates no interaction (i.e., there is simply an additive effect of both drugs) [44], Ψ<1 denotes drug synergy, and Ψ>1 indicates infra-additivity (or antagonism) between the two drugs. The schematic for the model can be found in Figure 4A.

For all model simulations and data-fitting, cell viability was calculated as

(9) $$Cell\;viability\left(t\right)=\frac{K\left(t\right)}{K\left(t\right)+A\left(t\right)}.$$

All simulations of the model were performed in Matlab R2019b using *ode45*. Fitting algorithms were performed by *lsqnonlin*. To avoid overfitting, $\overset{¯}{\delta }$ was assumed equal to $\delta_{1}\delta_{2}$ during fitting. The genetic algorithm optimisation routine was implemented using Matlab’s genetic algorithm function *ga* [45]. The objective function used for the genetic algorithm was

(10) $$\underset{D_{1}\left(0\right),D_{2}\left(0\right)}{\min}J(D_{1}\left(0\right),D_{2}\left(0\right)=\frac{K\left(48\right)}{K\left(48\right)+A\left(48\right)}+\frac{D_{1}\left(0\right)}{MTD_{1}}+\frac{D_{2}\left(0\right)}{MTD_{2}},$$

where $MTD_{1}$ and $MTD_{2}$ are the maximum tolerated dosages for the two drugs considered.

### 4.6. Statistical Analysis

Unless otherwise indicated, experiments were independently repeated at least 3 times, each time using duplicate or triplicate wells. GraphPad Prism (version 8) was used for statistical analysis of viability data. Drug synergy was calculated using viability data processed by the Matlab software plugin module Combenefit [32]. Statistical significance was calculated using either one-way analysis of variance (ANOVA) or Student’s *t*-test.

## Figures and Tables

**Table 1:** Interaction strength Ψ values for PAC-1 drug combinations, fitting the mathematical model (Figure 3A) to the dose–response measurements in Figure 2.

Drug	Ψ	Residual ^a^	Min Cell Viability ^b^
PAC-1 + Carboplatin	1.6	0.0015	0.4
PAC1 + Embelin	1.4	0.0073	0.16
PAC1 + Gemcitabine	1.8	0.0055	0.32
PAC1 + TRAIL	0.8	0.0050	0.14

^a^ normalized residual (sum of all residuals divided by the number of sample points); ^b^ minimum cell viability predicted by the model for the two-way dose response.

## Supplementary Materials

The following are available online at https://www.mdpi.com/article/10.3390/ijms22094699/s1, Figure S1: Proposed mechanism of procaspase-3 activation by PAC-1; Figure S2: Partial reduction of caspase-3 level is associated with reduced sensitivity of KGN cells to PAC-1; Figure S3: KGN cells express death receptor DR5 but not death receptor DR4; Figure S4: PAC-1 shows little synergy with radiotherapy; Figure S5: PAC-1 and TRAIL synergy reflected in mutual lowering of EC50 values; Figure S6: PAC-1 combined with TRAIL is less toxic to non-cancerous cells; Figure S7: KGN cell proliferation; Figure S8: Mathematical modelling calibrated to single drug dose-response curves at 24h and 48h; Figure S9: Enlarged panel from Main Text Figure 3D (left); Figure S10: Enlarged panel from Main Text Figure 3D (right); Figure S11: Quantifying the impact of risk and reward of increasing dosage; Figure S12: Caspase-3 and caspase-9 cleavage increases after treatment with PAC-1 and TRAIL in combination; Table S1: Model parameters (Equation (1) and Equations (2)–(9)) obtained for single-drug dose-response curves by fitting to 24 h and 48 h dose-response curves; Supplemental Materials and Methods with references.

## Abbreviations

AGCT	adult GCT
ANOVA	one-way analysis of variance
BID	BH3 interacting-domain death agonist
DAPI	4′,6-diamidino-2-phenylindole
DMEM	Dulbecco’s modified Eagle’s medium
DR	death receptor
FBS	fetal bovine serum
FITC	fluorescein isothiocyanate
GCT	granulosa cell tumor
IETD^175^	Ile-Glu-Thr-Asp175
MTD	Maximum tolerated dose
PAC-1	procaspase-activating compound 1
PAGE	polyacrylamide gel electrophoresis
PBS	phosphate-buffered saline
RFU	relative fluorescence units
RIPA	radio-immunoprecipitation assay
SDS	sodium dodecyl sulfate
shRNA	short hairpin RNA
SMAC	second mitochondria-derived activator of caspase
TRAIL	tumor necrosis factor-related apoptosis-inducing ligand
XIAP	X-linked inhibitor of apoptosis
