# Supplementary material for: Procaspase-Activating Compound-1 Synergizes with TRAIL to Induce Apoptosis in Established Granulosa Cell Tumor Cell Line (KGN) and Explanted Patient Granulosa Cell Tumor Cells In Vitro
Source: Int J Mol Sci. 2021 Apr 29;22(9):4699. doi: 10.3390/ijms22094699 (PMC8124867; doi:10.3390/ijms22094699)
Supplement: Supplementary file 1 [file ijms-22-04699-s001.zip › ijms-1159496-sup proof done.pdf]

1. Supplemental Figures and Tables

Table S1. Model parameters (Eq. (1) and Eq. (2)-(9)) obtained for single-drug dose-response curves by fitting to 24 h and 48 h dose-response curves (Figure S8).

**Drug**	IC_{50} (μM or ng mL^{-1})	γ (dimensionless)	δ (1/hour)	κ (1/hour)	I_{max} (dimensionless)
PAC-1 (μM)	3	1.35	0.04	4×10^{-5}	0.8764
Carboplatin (μM)	222	1.387	0.01	0.06	0.389
Gemcitabine (μM)	297	0.518	0.06	0.00012	0.768
Embelin (μM)	4	3.43	0.33	0.0157	0.930
TRAIL (ng mL^{-1})	5	0.874	0.11	0.0376	0.438

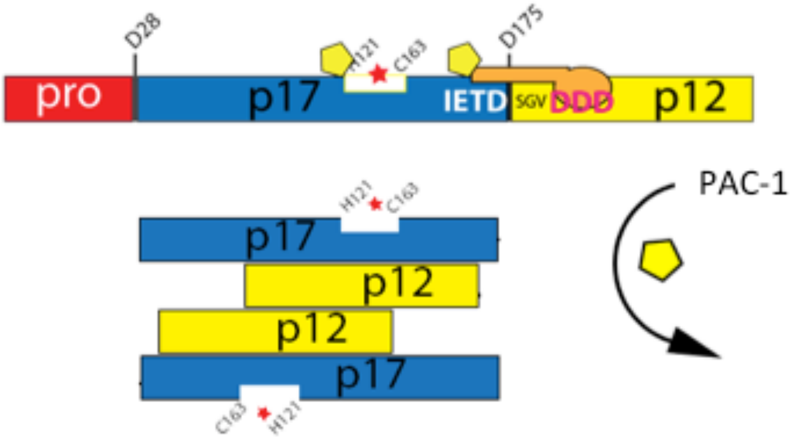

Figure S1. Proposed mechanism of procaspase-3 activation by PAC-1. Procaspase-3 is inhibited by Zn^{2+} ions (yellow pentagons) near its active site (red star) and triaspartic acid 'safety catch' (DDD). The cleavage site in procaspase-3, Ile-Glu-Thr-Asp175 (IETD175), generates a large/small subunit (p17/p12) dimer, with the fully mature caspase-3 forming a heterotetramer with two catalytically active sites. Model based on [1, 2].

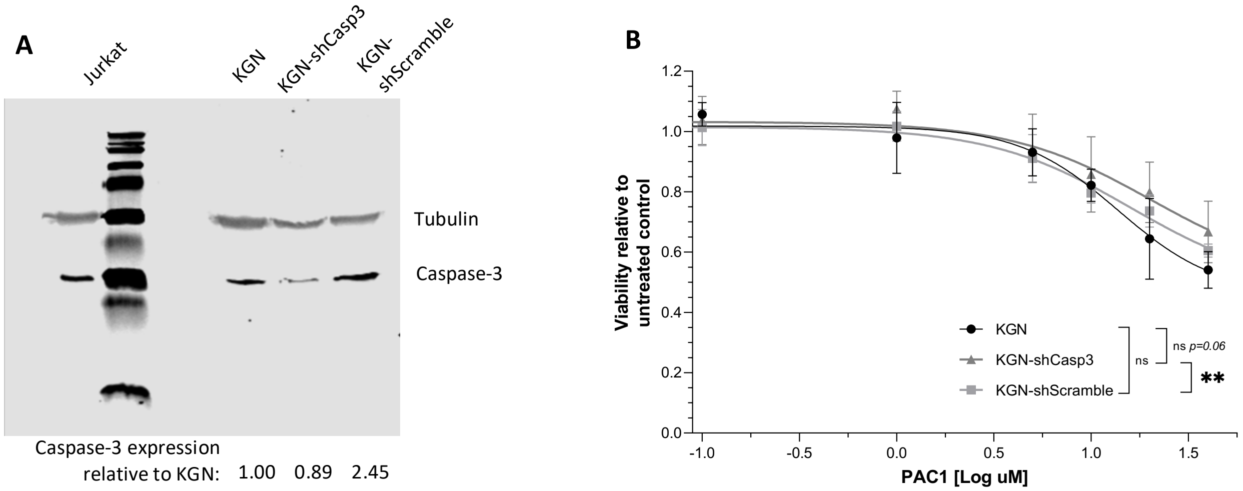

Figure S2. Partial reduction of caspase-3 level is associated with reduced sensitivity of KGN cells to PAC-1. **(A)** Left panels: Caspase-3 expression was detected by Western blot analysis of lysates from untreated KGN, KGN-shCasp3 (KGN stably transfected with CASP3 shRNA) and KGN-shScramble cells, with untreated Jurkat cell extract (Cell Signaling) as a positive control. Caspase-3 expression was quantified, then normalized to tubulin expression for each cell line. Normalized procaspase-3 levels from KGN, KGN-shCasp3 and KGN-shScramble cells are shown relative to that in KGN cells. (n = 1). **(B)** The indicated cells were treated with PAC-1 (0.1 – 40 μM) then viability determined 48 h later by resazurin metabolic assay. Data are normalized to untreated cells. Points represent mean +/- standard error of the mean (SEM) for 3 independent experiments. (** p<0.01).

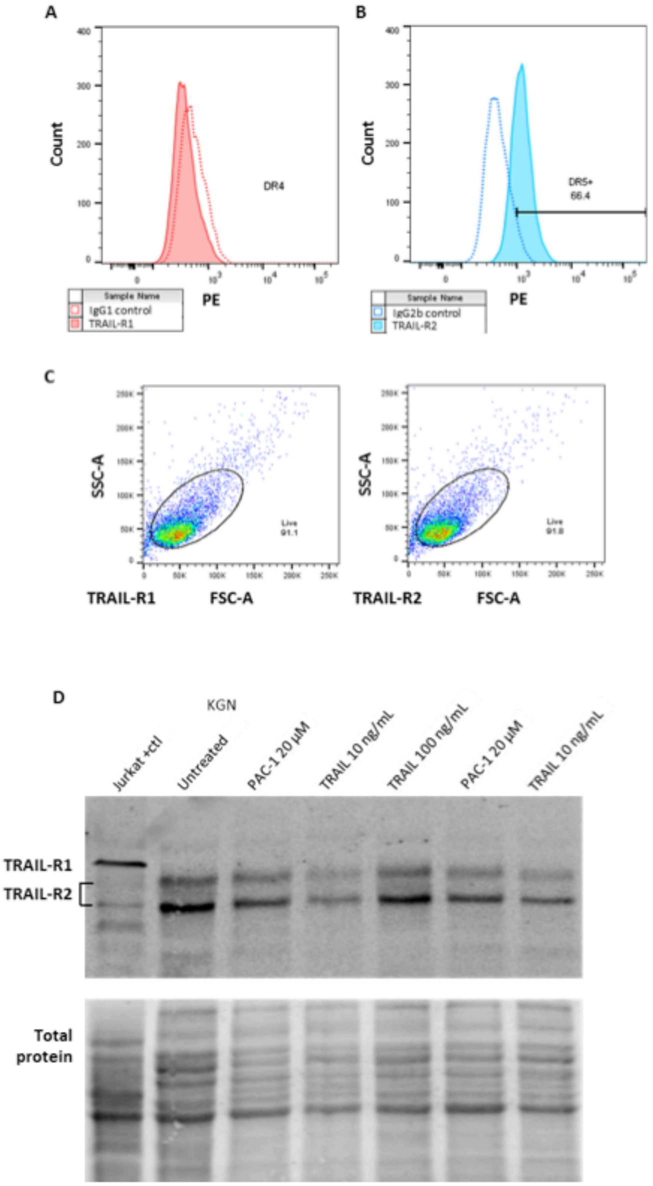

Figure S3. KGN cells express death receptor DR5 but not death receptor DR4. Untreated KGN cells were harvested and stained with PE-conjugated antibodies against DR4 (TRAIL-R1), DR5 (TRAIL-R2) or isotype controls (mouse IgG1 or IgG2B) as described in Supplemental Methods. Flow cytometry was performed on a BD Biosciences Fortessa X-20 and data were analysed using FlowJo version 10.5.3. (A) DR4 expression level compared to isotype control; (B) DR5 expression level compared to isotype control; (C) Gating strategy used to select population to be analysed; (D) Whole-cell lysate from indicated cell lines was probed for TRAIL-R1 (DR4) and TRAIL-R2 (DR5) (top panel) and stained for total protein using Licor RevertTM protein stain (bottom panel). Note that PAC-1 treatment does not increase the level of DR5 in KGN cells.

A

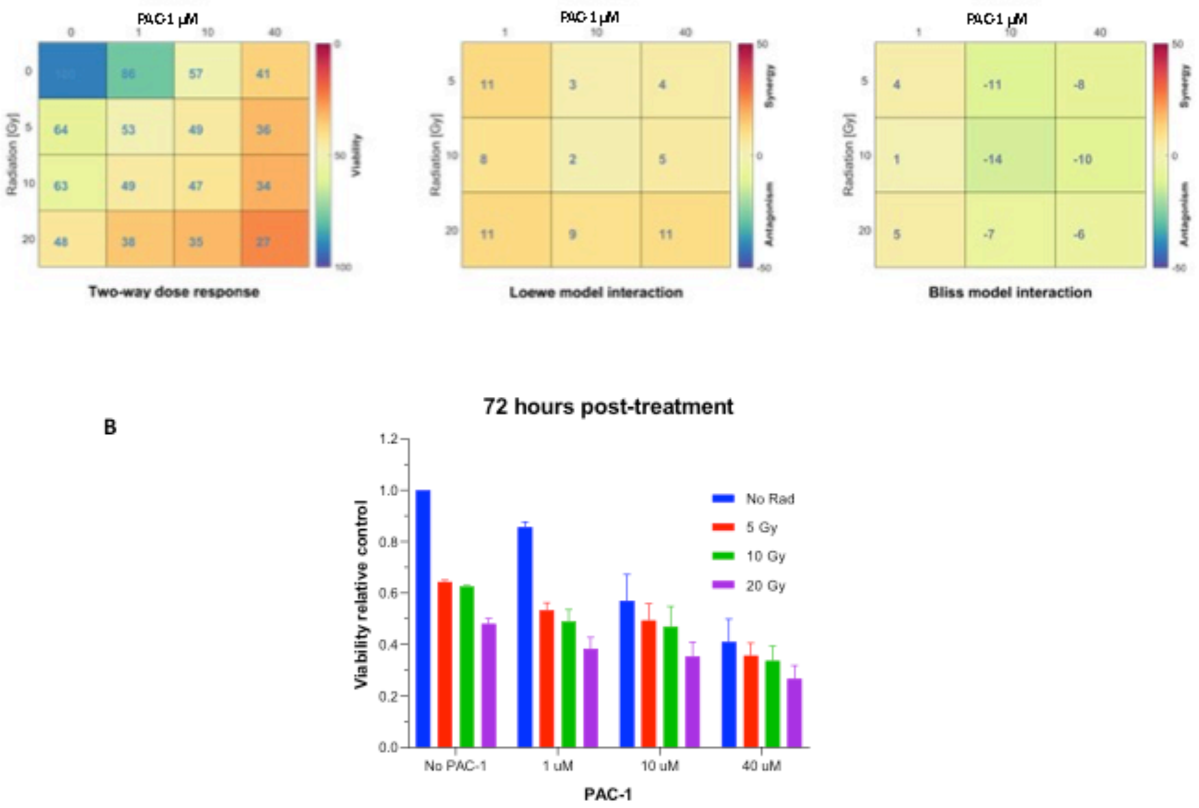

C

P-values adding PAC-1 to radiation[1]

		PAC-1 [μM]			
		0	1	10	40
Radiation [Gy]	0		ns	<.0001	<.0001
	5		ns	ns	0.0018
	10		ns	ns	0.0016
	20		ns	ns	0.0222

P-values adding radiation to PAC-1[2]

		Radiation [Gy]			
		0	5	10	20
PAC-1 [μM]	0		0.0001	<.0001	<.0001
	1		0.0005	<.0001	<.0001
	10		ns	ns	0.019
	40		ns	ns	ns

[1] – increasing dose of PAC-1 (compared to no PAC-1) at fixed dose of radiation
[2] – increasing dose of radiation (compared to no radiation) at fixed dose of PAC-1

Figure S4. PAC-1 shows little synergy with radiotherapy. KGN cells were treated with PAC-1 or left untreated, then immediately irradiated at the indicated doses as described in Supplemental Methods. (A) Viability and synergy were determined by resazurin metabolic assay 72 hours after irradiation. Viability was normalised to untreated control (normalized viability values indicated in the color scale) and synergy was analyzed with Matlab module Combenefit. (B) Viability data (re-plotted and analysed with GraphPad Prism) show a trend of increased killing with the combination compared to either agent alone, but the differences were not significant. (C) Adjusted p-values based on two-way analysis of variance for (1) increasing dose of PAC-1 (compared to no PAC-1) at fixed dose of radiation (left); and (2) increasing dose of radiation (compared to no radiation) at fixed dose of PAC-1 (right). (n=3)

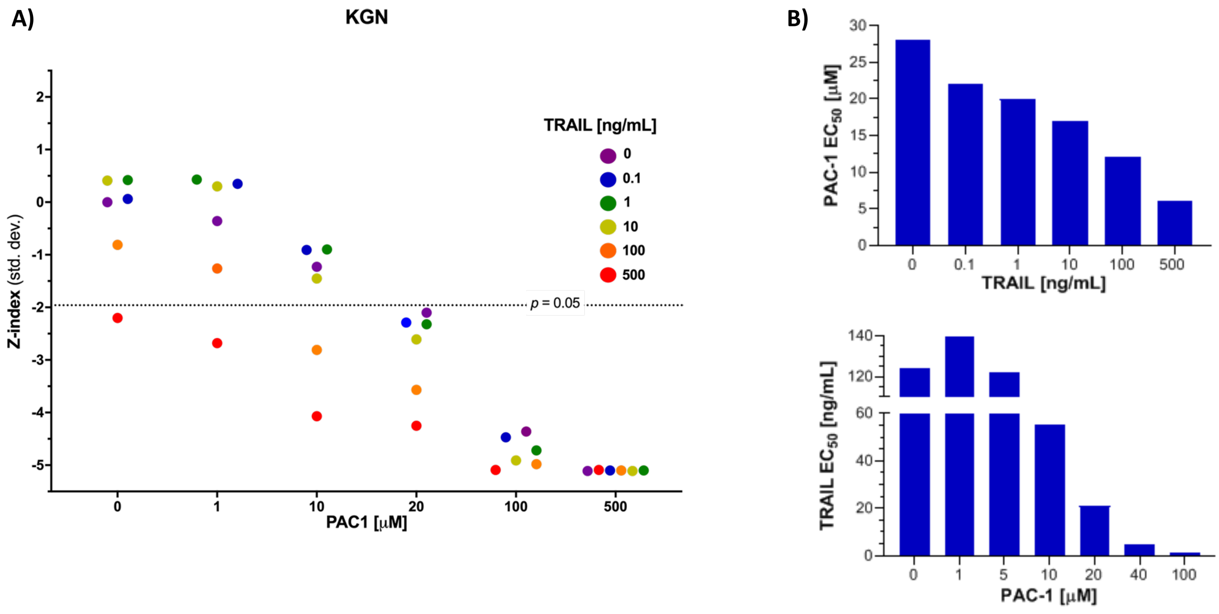

Figure S5. PAC-1 and TRAIL synergy reflected in mutual lowering of EC50 values. (A) 5000 cells/well in 96-well plates were treated in a two-way dose response assay with PAC-1 (0 – 500 μM) and TRAIL (0 – 500 ng/mL). After 48 hours viability was measured using a resazurin metabolic assay. Z-scores were calculated from viability data using the equation $Z = (Xo - \mu) / \sigma$ where Xo is the observed response in relative fluorescence units (RFU); μ is the mean RFU for untreated control wells, and σ is the standard deviation of untreated wells. A Z-score of >1.98 or < -1.98 indicates a significance of $p < 0.05$ [3]. (B) Using two-way dose-response viability data from panel (A), PAC-1 EC50 values were plotted against increasing doses of TRAIL (top panel), and TRAIL EC50 values were plotted against increasing doses of PAC-1 (bottom panel). (n = 3)

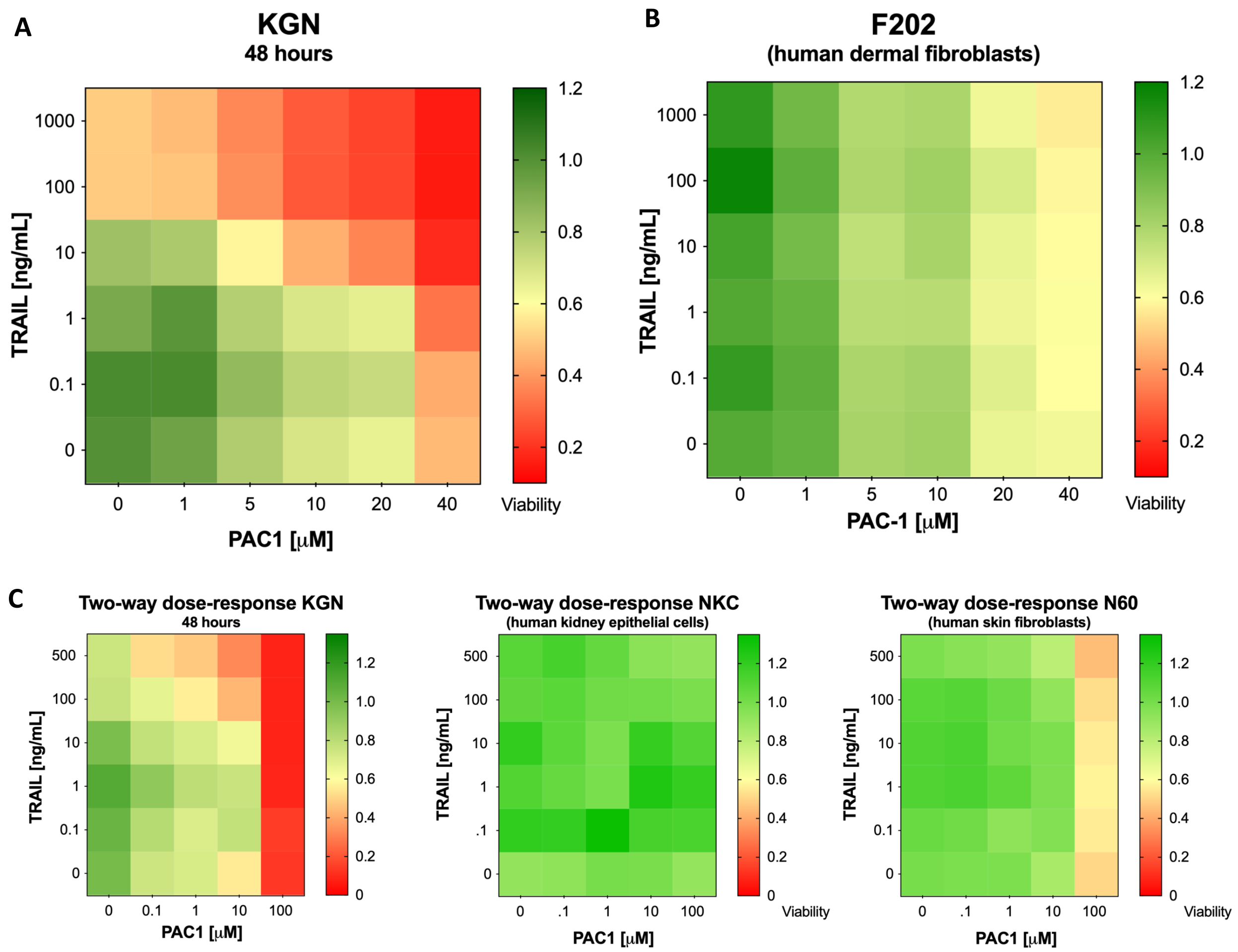

Figure S6. PAC-1 combined with TRAIL is less toxic to non-cancerous cells. Two-way dose-response assays were set up with 5000 cells/well in 96-well plates using **(A)** KGN cells; **(B)** normal human dermal fibroblasts (F202). Wells were treated for 48 h with TRAIL doses ranging from 0 – 1000 ng/mL combined with PAC-1 doses from 0 – 40 μM. Viability was determined by resazurin metabolic assay and normalized to untreated wells (normalized viability values indicated in the color scale). **(C)** Similar results were seen with a second fibroblast cell line (N60) and a cell line cultured from normal human kidney cells (NKC). (n = 3)

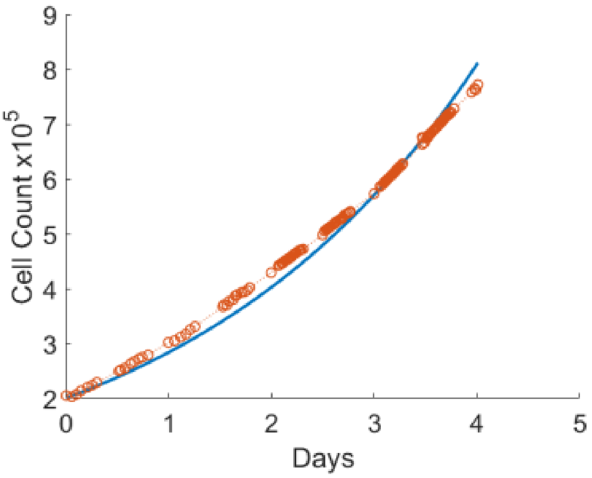

Figure S7. KGN cell proliferation. The proliferation rate of KGN cells was obtained by fitting an exponential growth curve (Eq. (2), blue curve) to KGN cell count measurements by Imai *et al*. [4] (orange dots).

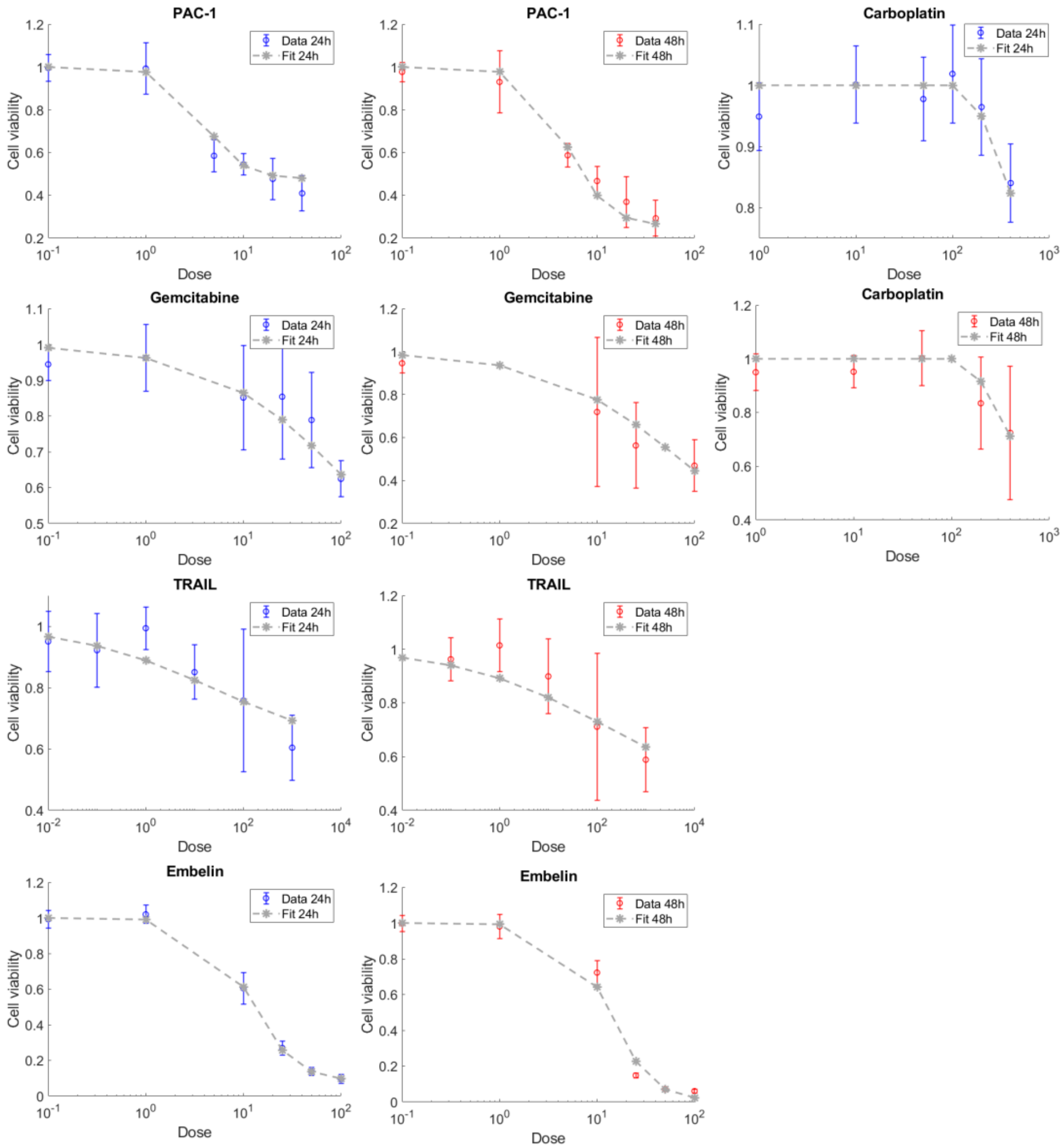

Figure S8. Mathematical modelling calibrated to single drug dose-response curves at 24h and 48h**.** The mathematical model was fit to single drug dose-response measurements for cell viability (Figure 2 Main Text) by fixing $D_2 = 0$ (no second drug). First, the cell viability measurements at 48h for each drug (PAC-1, Carboplatin, Gemcitabine, Embelin and TRAIL) were used to estimate IC_{50} and γ in the Imax curve (Eq. (1)). Then, fixing these values, the model (Eq. (2)-(9) with $Drug_2 = 0$ and $\Psi = 1$) was fit to the cell viability measurements at 24h and 48h simultaneously to estimate the drug-induced death rate of cells δ and drug decay rate κ. Measurements are plotted as mean and standard error bars for 24h (blue) and 48h (red). Overlaid is the model approximation (Eq. (1)-(8) with $D_2 = 0$) as a dashed line (grey) with stars representing the model approximation to each mean data point. Parameter values are given in Table S1.

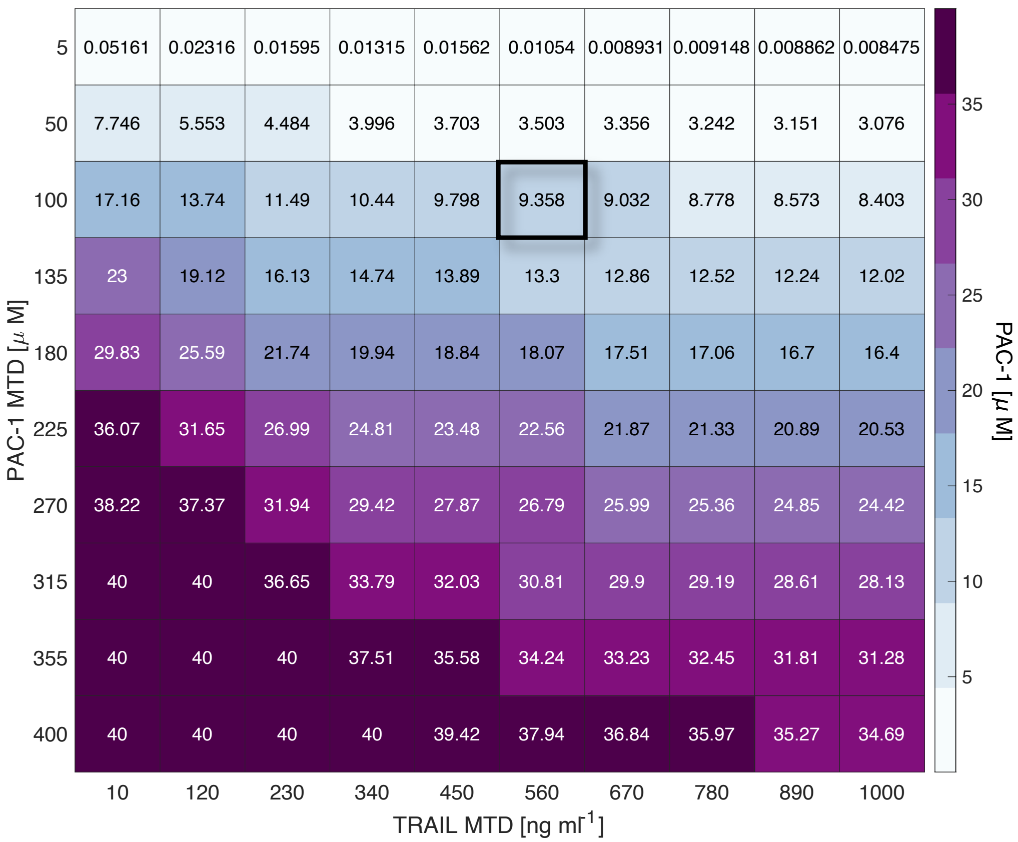

Figure S9. Enlarged panel from Main Text Figure 3D.

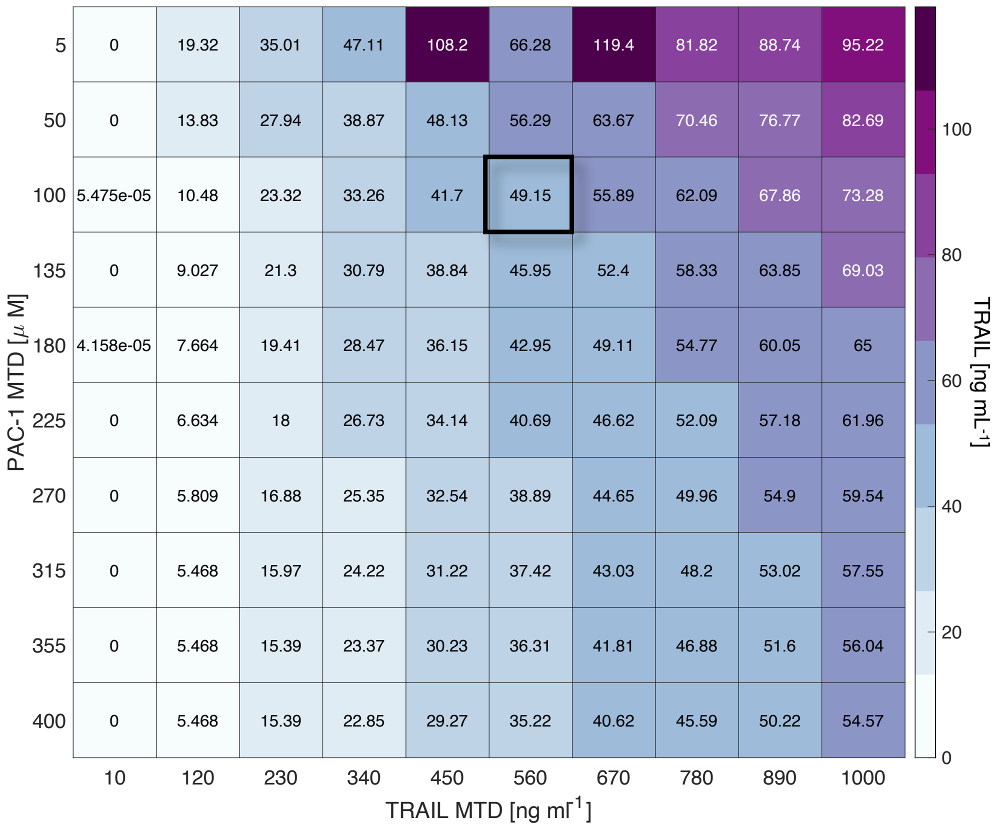

Figure S10. Enlarged panel from Main Text Figure 3D.

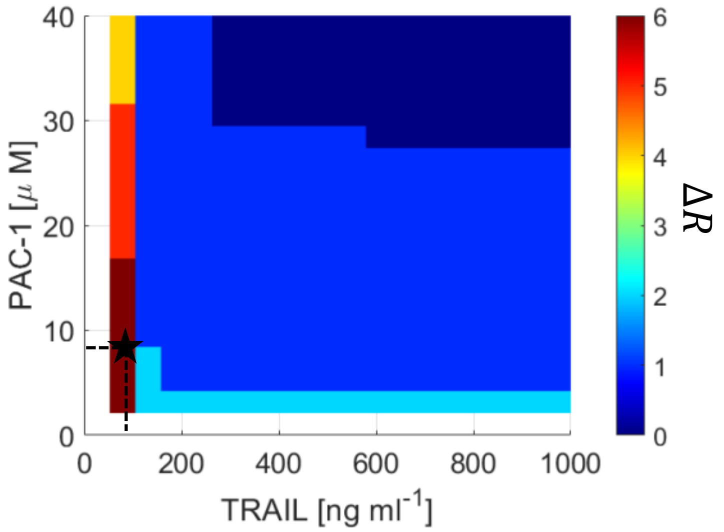

Figure S11. Quantifying the impact of risk and reward of increasing dosage. For small perturbations in the initial concentration of PAC-1 and TRAIL, we measured the change in cell viability relative to the increment in drug concentration ΔR. Regions are coloured based on the value of ΔR.

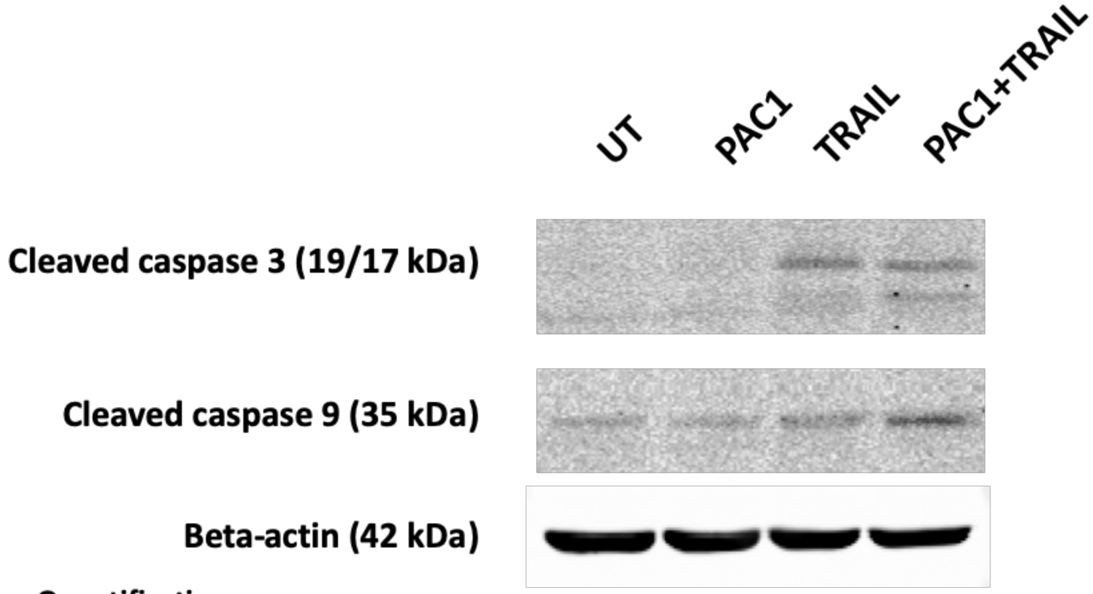

Quantification:

	UT	PAC1	TRAIL	PAC1+TRAIL
Cleaved casp-3 (19 kDa) mean:	1	1.23	3.71	3.64
(s.d.):		(0.61)	(0.91)	(1.2)
P-value (single agent vs. combination):		0.02	0.94	
Cleaved casp-3 (17 kDa) mean:	1	1.43	4.8	5.01
(s.d.):		(0.38)	(4.3)	(3.5)
P-value (single agent vs. combination):		0.13	0.95	
Cleaved casp-9 mean:	1	1.15	1.64	2.78
(s.d.):		(0.12)	(0.33)	(0.79)
P-value (single agent vs. combination):		0.01	0.06	

Figure S12. Caspase-3 and caspase-9 cleavage increases after treatment with PAC-1 and TRAIL in combination. KGN cells were treated with 20 μM PAC1, 10 ng/mL TRAIL or both for 24 h. The monolayer and supernatant were collected, lysed, and subjected to western blot analysis as described in the Supplemental Methods. The image is representative of four blots from two independent experiments. Cleaved caspase bands were quantified, normalized to beta-actin, and displayed relative to untreated (UT) samples (T-test used to determine significance).

2. Supplemental Materials and Methods

2.1. Generation of KGN-shCASP3 and KGN-shScramble cell lines

KGN-shCASP3 cells were generated by transfection with either plasmid pLKO.1-puro-U6-TRCN0000003549, -TRCN0000003550, or -TRCN0000003551 (Dharmacon), each expressing a different shRNA against CASP3. Following transfection, cells were selected with puromycin, then single-cell sorted and expanded, as in the manufacturer's protocol. Due to poor efficiency of transfection and cloning of KGN cells, we were successful in generating only three KGN-shCASP3 cell lines, one from each plasmid

construct. All three cell lines showed partially reduced caspase-3 expression by western blot analysis. The cell line with the most effective knock-down of caspase-3 (from shRNA TRCN0000003550) was selected for use in assays. KGN-shScramble cells were generated similarly by transfection with scramble shRNA which was a gift from David Sabatini (Addgene plasmid #1864; https://www.addgene.org/1864/).

2.2. Western blot analysis

Whole-cell protein extracts were prepared by collecting cells and medium from plates then pelleting by centrifugation at 4°C for 5 min at 500 x g. Cells were washed in cold PBS, re-centrifuged, then lysed on ice in RIPA buffer (150 mM NaCl, 50 mM Tris-HCl (pH 8.0), 0.5% sodium deoxycholate, 0.1% SDS, 1% NP-40, 0.1 mg/mL phenylmethylsulfonyl fluoride, and Halt protease inhibitor cocktail (Thermo Fisher). Lysates were clarified by centrifugation at 4°C for 20 min at 20,000 x g, then assayed for protein (BCA protein assay kit, Thermo Fisher). For western blots, up to 40 μg of protein was resolved by SDS-PAGE (12% (w/v) acrylamide for blots in Figure S2 and S3, 10% for Figure S12), then transferred to Immobilon-FL PVDF membranes (EMD Millipore). Chameleon Duo protein ladder (#928-60000) was purchased from Li-COR Biosciences. Protein transfer was confirmed by staining membranes with Revert™ Total Protein Stain (Li-COR Biosciences #926-11010), then washed before blocking with Odyssey blocking buffer (Li-COR Biosciences #927-50003) for 1 h at RT, and incubating overnight at 4°C with primary antibodies (diluted 1:1000 in blocking buffer with 0.2% Tween). Western blot antibodies for beta-tubulin (#2146), cleaved caspase-3 (#9661), cleaved caspase-9 (#20750), total caspase-3 (#9662) and TRAIL-R2 (#8074) as well as the Jurkat positive control lysate (#2043) were purchased from Cell Signaling. Antibody for human TRAIL-R1 (ab13890) was purchased from Abcam. Fluorescent-dye conjugated secondary antibodies (Li-COR Biosciences #926-68, #926-68070) were diluted in blocking buffer (1:20000) containing 0.2% Tween and 0.01% SDS, and incubated with the membranes at room temperature for 1 h. Washed membranes were scanned using an Odyssey scanner (Li-COR Biosciences) and the images analyzed using ImageStudio software (Li-COR Biosciences).

2.3. Irradiation of cells

15,000 KGN cells were seeded in 48-well plates, incubated overnight and then treated with either fresh medium or PAC-1 as indicated and irradiated (or mock-irradiated) in a Faxitron MultiRad 160 with one dose of either 5, 10 or 20 Gy. 72 h after irradiation, cells were assayed for viability using methodology described in section 2.2. ANOVA adjusted p value calculations were performed using GraphPad Prism with Tukey's multiple comparison test.

2.4. Flow cytometry

For each sample to be analysed, 2.5 x 10^5 cells were pelleted at 4°C for 5 min at 300 x g then washed in cold FACS buffer (PBS, 1% BSA, 1mM EDTA, 0.1% sodium azide) and re-centrifuged. Washed cells were resuspended in 5% serum and incubated at room temp for 15 min. Cells were then washed with FACS buffer and resuspended in 10 μL phycoerythrin- (PE-) conjugated antibodies (R&D Systems) against DR4 (TRAIL-R1, #FAB347P), DR5 (TRAIL-R2, #FAB6311P) or isotype controls (mouse IgG1 (#IC002P) or IgG2B (#IC0041P)) at the manufacturer's recommended dilutions. Cells were allowed to bind antibodies for 30 min on ice in the dark, then washed again with cold FACS buffer, resuspended in 0.5 mL cold FACS buffer, vortexed and strained to remove aggregates.

Cell suspensions were analyzed on an LSR-Fortessa X20 flow cytometer (BD Biosciences) using FACS DiVa software (BD Biosciences). Cell populations were gated using forward-scatter area and side-scatter area and a single cell population was gated by using forward-scatter height and forward-scatter area. PE signal was detected on the PE channel using a 561-nm laser. FlowJo software (Version 10.5.3) was used to perform analysis of cell membrane expression.

3. References

1. Daniel, A.G.; E.J. Peterson, E.J.; Farrell, N.P. The bioinorganic chemistry of apoptosis: potential inhibitory zinc binding sites in caspase-3. *Angew Chem Int Ed Engl* **2014,** *53,* 4098-4101.

2. Peterson, Q.P.; Goode, D.R.; West, D.C.; Ramsey, K.N.; Lee, J.J.; Hergenrother, P.J. PAC-1 activates procaspase-3 *in vitro* through relief of zinc-mediated inhibition. *J Mol Biol* **2009**, *388*, 144-158.

3. Birmingham, A.; Selfors, L.M.; Forster, T.; Wrobel, D.; Kennedy, C.J.; Shanks, E.; Santoyo-Lopez, J.; Dunican, D.J.; Long, A.; Kelleher, D.; Smith, Q.; Beijersbergen, R.L.; Ghazal, P.; Shamu, C.E. Statistical methods for analysis of high-throughput RNA interference screens. *Nat Methods* **2009**, *6*, 569-575.

4. Imai, M.; Muraki, M.; Takamatsu, K.; Saito, H.; Seiki, M.; Takahashi, Y. Spontaneous transformation of human granulosa cell tumours into an aggressive phenotype: a metastasis model cell line. *BMC Cancer* **2008**, *8*, 319.
